# Systematic Evaluation of Affinity Enrichment Methods for O-GlcNAc Proteomics

**DOI:** 10.1021/acs.jproteome.4c00388

**Published:** 2024-09-20

**Authors:** Chunyan Hou, Ci Wu, Zichun Wu, Yifan Cheng, Weiyu Li, Hui Sun, Junfeng Ma

**Affiliations:** † Department of Oncology, Lombardi Comprehensive Cancer Center, Georgetown University Medical Center, Washington, District of Columbia 20007, United States; ‡ Information Science and Technology College, Dalian Maritime University, Dalian 116026, China; § Department of Applied Mathematics and Statistics, Johns Hopkins University, Baltimore, Maryland 21218, United States; ∥ Department of Biochemistry, College of Life Sciences, Wuhan University, Wuhan 430072, China

**Keywords:** O-GlcNAc, proteomics, antibody, AANL6, OGA mutant, HCD-pd-EThcD, Sequest HT, Byonic, FragPipe

## Abstract

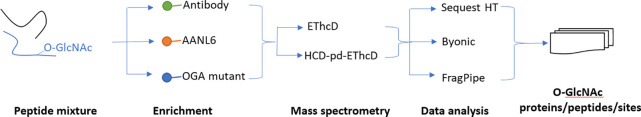

O-Linked β-*N*-acetylglucosamine (O-GlcNAc) modification (i.e., O-GlcNAcylation) on proteins plays critical roles in the regulation of diverse biological processes. However, protein O-GlcNAcylation analysis, especially at a large scale, has been a challenge. So far, a number of enrichment materials and methods have been developed for site-specific O-GlcNAc proteomics in different biological settings. Despite the presence of multiple methods, their performance for the O-GlcNAc proteomics is largely unclear. In this work, by using the lysates of PANC-1 cells (a pancreatic cancer cell line), we provided a head-to-head comparison of three affinity enrichment methods and materials (i.e., antibody, lectin AANL6, and an OGA mutant) for site-specific O-GlcNAc proteomics. The enriched peptides were analyzed by HCD product-dependent EThcD (i.e., HCD-pd-EThcD) mass spectrometry. The resulting data files were processed by three different data analysis packages (i.e., Sequest HT, Byonic, and FragPipe). Our data suggest that each method captures a subpopulation of the O-GlcNAc proteins. Besides the enrichment methods, we also observe complementarity between the different data analysis tools. Thus, combining different approaches holds promise for enhanced coverage of O-GlcNAc proteomics.

## Introduction

O-Linked β-*N*-acetylglucosamine (O-GlcNAc) modification (O-GlcNAcylation) is a dynamic and reversible post-translational modification that involves the addition or removal of a single sugar moiety, *N*-acetylglucosamine (GlcNAc), to serine or threonine residues on proteins.^1^ O-GlcNAc modification on proteins plays a crucial role in regulating protein function, cellular signaling, and gene expression.^2−6^ Thanks to technical advances, O-GlcNAc proteomics is emerging as a powerful tool for unraveling the complex regulatory mechanisms of O-GlcNAc modification in health and disease.^7−10^

Despite its functional importance, site-specific O-GlcNAcylation analysis has been challenging, partly due to the low abundance of the O-GlcNAc modifications in complex samples. Thus, O-GlcNAc enrichment is crucial for large-scale O-GlcNAc site mapping. The continuous endeavor in O-GlcNAc enrichment techniques has significantly advanced O-GlcNAc proteomics.^7−10^ So far, various methods have been developed to enrich the O-GlcNAc proteins/peptides. In general, two categories of enrichment methods and materials have been developed. In one strategy, O-GlcNAc proteins/peptides are subjected to (bio)chemical derivatization (e.g., alkaline β-elimination followed by Michael addition)^11,12^ and metabolic/chemoenzymatic labeling followed by biorthogonal reactions (e.g., click chemistry).^13−22^ In another strategy, affinity enrichment is performed on native O-GlcNAc proteins/peptides. As a promising strategy, several affinity enrichment techniques have been developed. For example, sequential lectin weak affinity chromatography (LWAC, e.g., by using a 2 mm × 250 mm stainless steel column packed with Wheat germ agglutinin) was adopted.^23−27^ Due to the weak binding affinity, multiple rounds of enrichment (retention) are usually needed to obtain GlcNAc-enriched fractions for analysis. Several types of antibodies have also been tentatively explored for O-GlcNAc enrichment in O-GlcNAc proteomics studies.^28−32^ AANL, a lectin isolated from the mushroom *Agrocybe aegerita*, was found to have a higher affinity toward the terminal nonreducing GlcNAc than other GlcNAc-binding lectins.^33^ Among its mutants, AANL6, which contains six carbohydrate binding sites (with the same amino acid sequences), showed improved specificity.^34^ Besides, a mutant of OGA, the only enzyme that removes O-GlcNAc from proteins, was used for the enrichment of O-GlcNAc peptides.^35^ With the catalytic site 298 aspartic acid (D) mutated to asparagine (N), the inactive*Clostridium perfringens* NagJ (CpOGA^D298N^) retains the ability to bind to O-GlcNAylated peptides.^36^ Targeting the hydrophilicity of the glycan on peptides, hydrophilic interaction chromatography was also used to enrich O-GlcNAc peptides.^37,38^

Besides enrichment methods, great efforts in data acquisition and data analysis have been made. High-resolution mass spectrometers coupled with advanced fragmentation techniques, especially the combination of electron transfer dissociation (ETD) and higher-energy collisional dissociation (HCD), have enabled the site mapping of labile glycosylation with greater accuracy and confidence.^39,40^ EThcD incorporates additional HCD fragmentation following the ETD step, generating a higher number of product ions and improving peptide sequencing. The HCD product-dependent EThcD (HCD-pd-EThcD) selectively fragments precursors that generate the HexNAc oxonium ions in the HCD scan, allowing targeted analysis of O-GlcNAc-modified peptides. In addition, a number of search engines have been applied for O-GlcNAc proteomics data analysis, e.g., Sequest HT,^41,42^ Byonic,^43,44^ and MSFragger (FragPipe).^45,46^ Since different algorithms and scoring approaches are used, varied performance and capabilities might be anticipated. Last but not least, to distinguish the isobaric HexNAc modifications (e.g., O-GlcNAc and Tn antigens) on proteins, empirical and computational models have been proposed to facilitate glycoproteomics data analysis.^40,47^

Of note, analytical methods, especially the enrichment methods/materials, were applied for different kinds of samples, and different starting amounts of total proteins were employed for O-GlcNAc proteomics. Providing the huge differences in the O-GlcNAc level among different sample sources,^6^ it is hard to compare the performance of different approaches. In this study, we performed a head-to-head evaluation of different methods (especially affinity enrichment methods/materials) for O-GlcNAc proteomics, given that such a performance comparison is lacking. In brief, by using the lysates of PANC-1 (a pancreatic cancer cell line), we compared three affinity enrichment methods/materials (i.e., antibody, lectin AANL6, and the OGA mutant CpOGA^D298N^) for O-GlcNAc proteomics. The enriched peptides were analyzed by two different mass spectrometric approaches: EThcD or HCD-pd-EThcD. The resulting data files were then processed using three different data analysis packages (i.e., Sequest HT, Byonic, and FragPipe), followed by HexNAcQuest validation.

## Experimental Section

### Materials and Reagents

Two recombinant proteins, AANL6 (2.72 mg/mL, 85%) and the OGA mutant CpOGA^D298N^ (0.39 mg/mL, 90%), were customized by GenScript (Piscataway, NJ). The PTMScan O-GlcNAc [GlcNAc-S/T] motif kit (including immunoaffinity beads and 10× IAP buffer) was bought from Cell Signaling Technology Inc. (Danvers, MA). The AminoLink coupling resin, cyanoborohydride (NaCNBH_3_), formic acid (FA, LC/MS grade), phosphate-buffer saline tablets, and a BCA protein assay kit were obtained from Thermo Fisher Scientific (Waltham, MA). 1,4-Dithiothreitol (DTT) was bought from MP Biomedicals (Solon, OH). Iodoacetamide (IAA) and LC/MS grade reagents of water, methanol, and acetonitrile (ACN) were ordered from VWR (Radnor, PA). PUGNAc, triethylammonium bicarbonate (TEAB) buffer (1 M, pH 8.5), phosphoric acid (≥85 wt % in H_2_O), Trizma base, Tris-buffered saline (TBS, 10×), sodium dodecyl sulfate (SDS), Dulbecco’s modified Eagle’s medium (DMEM), penicillin–streptomycin (solution stabilized, 10,000 units penicillin and 10 mg streptomycin/mL), cOmplete EDTA-free protease inhibitor cocktail tablets, and trypsin from porcine pancreas were purchased from Sigma-Aldrich (St. Louis, MO). Benzonase nuclease was obtained from MilliporeSigma (Burlington, MA). Bovine calf serum (BCS) was ordered from Cytiva (Marlborough, MA). Thiamet G (TMG) was bought from Cayman Chemical (Ann Arbor, MI). Hydrochloric acid (HCl, 5.00 N) was from RICCA Chemical (Arlington, TX). LC/MS grade solutions of 0.1% FA in H_2_O and 0.1% FA in ACN were ordered from Honeywell (Charlotte, NC). S-Trap midi columns were obtained from ProtiFi (Fairport, NY). A mixture of proteotypic O-GlcNAc peptides (SpikeMix PTM-Kit 57) was obtained from JPT Peptide Technologies GmbH (Berlin, Germany). MS-compatible yeast protein extracts were bought from Promega (Madison, WI).

### Immobilization of AANL6 and OGA Mutant

To prepare the AANL6 immobilized beads, 0.5 mL of the AminoLink slurry was washed with 1 mL of coupling buffer (1× PBS, pH 7.4) 3 times. After the removal of the supernatant, the beads were incubated with 0.46 mg of AANL6, 1.2 mL of the coupling buffer, and 0.07 mL of 1 M NaCNBH_3_ on a rotator at 4 °C overnight. The supernatants before and after incubation were saved to estimate the immobilized amount of AANL6. The beads were then washed with 1 mL of 1 M Tris-HCl buffer (pH 8) 3 times. One milliliter of 1 M Tris-HCl buffer (pH 8) and 0.07 mL of 1 M NaCNBH_3_ were added to incubate with the beads at 4 °C for 0.5 h. Finally, the beads were washed with 1× TBS 3 times and saved at 4 °C before use.

The immobilization of the OGA mutant (CpOGA^D298N^) was similar to the above process, except that the pH of the coupling buffer was adjusted to 4.7 to improve the efficiency. Specifically, 0.35 mg of the OGA mutant, 0.4 mL of coupling buffer, and 0.07 mL of 1 M NaCNBH_3_ were incubated with the AminoLink beads.

### Cell Culture, Protein Extraction, and Protein Digestion

The pancreatic ductal cell line PANC-1 cells were cultured as reported previously.^38^ In brief, PANC-1 cells grown in DMEM supplemented with 10% BCS and 1% penicillin–streptomycin were maintained in a 37 °C incubator with 5% CO_2_ and treated with 2 μM TMG for 4 h before harvesting.

PANC-1 cell pellets were suspended and incubated in 200 μL of cell lysis buffer containing 5% SDS, 2 μM PUGNAc, 1× protease inhibitor cocktail, 50 mM TEAB, 2 μL of Benzonase, and 1 mM MgCl_2_. The cell suspension was then sonicated with a probe-tip sonicator for three pulses (10 s on and 20 s off for each pulse) on ice. The cell lysate was centrifuged at 13,000*g* for 15 min at 4 °C with the supernatant saved and the protein concentration estimated by the BCA assay.

Equal amounts of extracted proteins were processed with the suspension trapping (S-Trap) method as described previously with minor modifications.^48,49^ Briefly, the proteins were sequentially reduced in 20 mM DTT, alkylated in 40 mM IAA, acidified with ∼1% phosphoric acid, and diluted with six volumes of the S-Trap buffer (90% aqueous methanol in 100 mM TEAB, pH 7.5). The mixture was loaded onto a midi S-Trap column and washed with the S-Trap buffer 3 times. Then, trypsin was loaded onto the column at a protease to substrate mass ratio of 1:20 in 50 mM TEAB. After incubation at 37 °C overnight, the same amount of trypsin was added for a second treatment of 4 h at 37 °C. The resulting peptides were eluted by 500 μL of 50 mM TEAB, 0.2% formic acid, and 50% ACN sequentially. The eluates were combined and dried with a lyophilizer.

### Enrichment of O-GlcNAc Peptides

The lyophilized peptides (10 mg) were dissolved in 1× IAP buffer and centrifuged at 10,000*g* for 5 min at 4 °C. The supernatant was enriched with PTMScan O-GlcNAc [GlcNAc-S/T] immunoaffinity beads, AANL6 immobilized beads, and OGA mutant immobilized beads, respectively. The eluates (after enrichment) were purified with a homemade C8 ZipTip to remove residue beads and dried down with a lyophilizer.

The O-GlcNAc immunoaffinity enrichment was performed according to the manufacturer’s instructions with minor modifications. Briefly, 160 μL of the slurry was washed with 1 mL of PBS 4 times and incubated with the peptide solution on a rotator at 4 °C for 2 h. After the removal of the supernatant, the beads were washed with 0.5 mL of IAP buffer and 1 mL of chilled water 4 times, respectively. Finally, 100 μL of 0.15% trifluoroacetic acid (TFA) was added to elute the enriched peptides. This step was repeated once. As a comparison, we also performed fractionation and then enrichment. To each of the 12 high-pH HPLC fractions obtained (for, 20 μL of the slurry was added and incubated at 4 °C for 2 h. After removal of the supernatant, the beads were washed sequentially with 0.5 mL of IAP buffer and 1 mL of chilled water 4 times, with enriched peptides eluted by 55 μL of 0.15% TFA twice.

The enrichment using AANL6 or OGA mutant immobilized beads was performed based on previously described steps^34,36,50^ with slight modifications. In brief, 875 μL of the AANL6 slurry (with 0.81 mg of AANL6 immobilized) and 875 μL of the OGA mutant slurry (with 0.22 mg of the OGA mutant immobilized) were washed with TBS buffer and incubated with the protein digests on a rotator at 4 °C overnight. The beads were then washed with 4 mL of TBS and 4 mL of H_2_O 3 times, respectively. At last, the enriched peptides were eluted by 1 mL of 0.15% TFA, 0.5 mL of 0.15% TFA, and 0.5 mL of 50% ACN sequentially.

### High-pH Reversed-Phase Fractionation

To decrease sample complexity, high-pH RPLC fractionation of peptides was performed, similar to those described previously.^38,51^ The lyophilized peptides before or after enrichment were fractionated with an Acquity UPLC system coupled to a tunable ultraviolet (UV) detector (Waters, Milford, MA). The peptides resuspended in 3 mM ammonium hydroxide solution were separated on an XBridge C18 column (5 μm, 4.6 mm × 25 cm) at room temperature. The flow rate was set at 1 mL/min. A 60 min gradient of mobile phase A (3 mM ammonium hydroxide in water) and mobile phase B (3 mM ammonium hydroxide in 90% ACN) was used for separation: 0 min, 1% B; 2 min, 1% B; 5 min, 5% B; 40 min, 35% B; 43 min, 90%; 45 min, 90% B; 50 min, 2% B; 60 min, 2% B. The UV detection wavelength was set at 214 nm, and the 1 min fractions were collected manually for the first 48 min. The 48 fractions were then concatenated into 12 fractions at an interval of 12 and dried on a lyophilizer.

### NanoUPLC-MS/MS

The enriched O-GlcNAc peptides were analyzed using a nanoAcquity UPLC system (Waters) coupled with an Orbitrap Fusion Lumos mass spectrometer (Thermo Fisher) with similar settings described previously.^38^ Samples were resuspended in 0.1% FA solution and loaded onto a C18 Trap column (Waters Acquity UPLC M-Class Trap, Symmetry C18, 100 Å, 5 μm, 180 μm × 20 mm) at 10 μL/min for 4 min. At a flow rate of 400 nL/min, the peptides were then separated on an analytical column (Waters Acquity UPLC M-Class, peptide BEH C18 column, 300 Å, 1.7 μm, 75 μm × 250 mm) at 45 °C. A 150 min gradient of buffer A (2% ACN, 0.1% formic acid) and buffer B (0.1% formic acid in ACN) was used for separation: 0 min, 1% B; 1 min, 5% B; 120 min, 22% B; 130 min, 36% B; 135 min, 50% B; 140 min, 90% B; 145 min, 90% B; 145.1 min, 1% B; 150 min, 1% B. The Orbitrap Fusion Lumos mass spectrometer was performed in data-dependent acquisition (DDA) mode. The ion spray voltage and ion transfer temperature were set at 2.4 kV and 275 °C, respectively. The mass spectra were recorded with Xcalibur 4.0 under the following conditions: Mass range: 375–1500 *m*/*z*; Orbitrap Resolution: 120,000; Scan Range: 375–1500 *m*/*z*; RF Lens: 30%; AGC Target: Standard; Maximum Injection Time Mode: Auto; Microscans: 1; Charge state: 2–7; Exclusion duration: 40 s; Cycle Time: 3 s. EThcD or HCD-pd-EThcD was used for the MS/MS acquisition. In the HCD-pd-EThcD mode, EThcD was triggered with five fragment peaks (i.e., *m*/*z* 204.0867, 138.0545, 126.055, 186.0761, and 168.0655) observed in the HCD scan. MS/MS parameters were set as below: Isolation Mode: Quadrupole; Isolation Window: 1.6 *m*/*z*; Stepped Collision Energy: 22.5, 30, and 37.5%; Resolution: 30,000; Normalized AGC Target: 200%. Supplemental activation (SA) collision energy of EThcD was set as 30%.

### Data Analysis

Three different engines were used to search the raw data files against the UniProt *Homo sapiens* database (TaxID 9606, Released on 2020–07–20, 20,353 sequences). The Sequest HT and Byonic nodes were run in the Proteome Discoverer (Thermo Fisher Scientific, version 2.4) with the following settings: full tryptic digestion and up to two missed cleavages; the precursor mass tolerance was set at 10 ppm, whereas the fragment-mass tolerance was set at 0.02 Da. Carbamidomethylation of cysteines (+57.0215 Da) was set as a fixed modification, and variable modifications of the O-GlcNAc (S or T, +203.079 Da), acetyl (N-terminus, +42.011 Da), and methionine oxidation (M, +15.9949 Da), loss (M, −131.040 Da), or loss + acylation (M, −89.030 Da) were allowed. The maximum dynamic modification number was five. The false-discovery rate (FDR) was determined using a target-decoy search strategy. The decoy-sequence database contains each sequence in reverse orientations, enabling FDR estimation. The corresponding FDRs at the PSM, peptide, and protein levels were all less than 1%. The modification sites identified by Sequest HT were further filtered by IMP-ptmRS with a site probability ≥0.75. The modification sites identified by Byonic were filtered by the delta mod score of not less than 10. FragPipe (version 19.0) with the MSFragger engine and glyco-O-Pair workflow performed with the default settings. The variable modifications were 15.9949 Da on M, 42.0106 Da on N-terminal, and 57.02146 Da on C, with five variable modifications at maximum. A cutoff of the site probability at 0.75 was applied for the modified sites. The glycan site composition was set to N1 for all PSMs.

All PSMs corresponding to HexNAc modification sites were processed by HexNAcQuest^47^ to distinguish O-GlcNAc and O-GalNAc modifications. The O-GalNAc PSMs identified by HexNAcQuest were removed from the final results.

The R packages “tidyverse”,^52^ “tidysq”,^53^ “ggseqlogo”,^54^ and “VennDiagram”^55^ were used for data analysis and plotting. The Database for Annotation, Visualization, and Integrated Discovery (DAVID)^56^ was used for the enrichment of gene ontology (GO) terms.

## Results and Discussion

### Experimental Design for Systematic Evaluation of Factors Influencing O-GlcNAc Proteomics

Given the lack of evaluation of different analytical procedures (especially enrichment methods/materials) for O-GlcNAc proteomics, a systematic comparison was conducted in this work. Due to the low levels of O-GlcNAc modification in complex samples, selective enrichment of O-GlcNAc peptides (usually in combination with fractionation) is generally needed prior to their analysis by tandem mass spectrometry. Moreover, whether enriching the O-GlcNAc peptides before or after HPLC fractionation would affect the final results is largely unknown.

**Figure 1:**
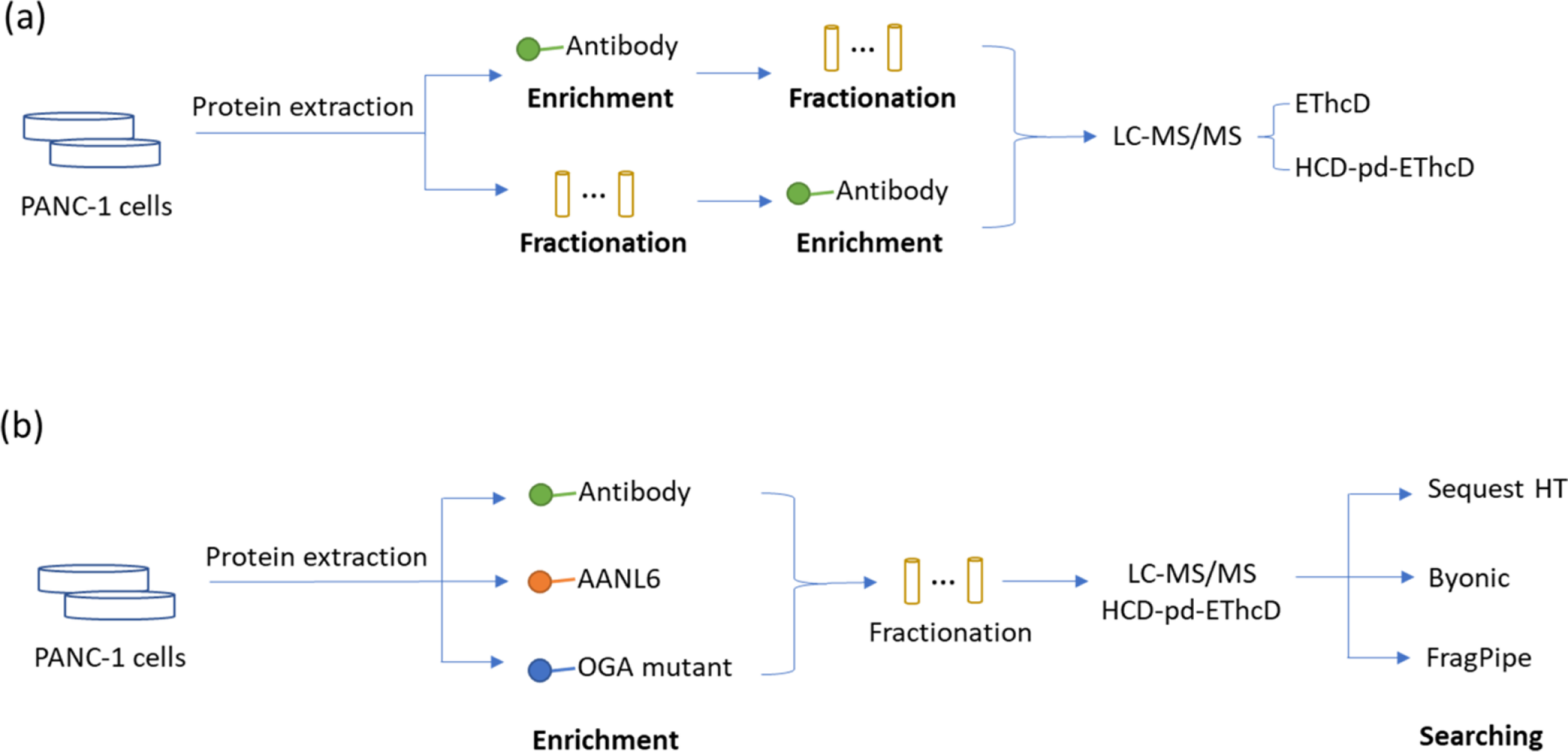
Scheme of protein O-GlcNAc proteomics analyzed with different workflows. (a) Antibody-based analytical approaches (either fractionation followed by enrichment or enrichment followed by fractionation). (b) Enrichment with three materials (followed by fractionation), HCD-pd-EThcD mass spectrometry, and data analysis by using three different search engines.

By using PANC-1 cell lysates, we first compared two enrichment/fractionation workflows by using the immobilized O-GlcNAc [GlcNAc-S/T] motif antibody beads: (1) enrich O-GlcNAc peptides from the protein digests and then fractionate into multiple fractions and (2) fractionate the protein digests and then enrich O-GlcNAc peptides from each fraction (Figure 1a). To obtain the site-specific information on the enriched O-GlcNAc peptides, two different MS/MS fragmentation methods (i.e., EThcD and HCD-pd-EThcD) were used (Figure 1a). We then tested the enrichment performance of two other methods/materials: AANL6 and an OGA mutant (CpOGA^D298N^) (Figure 1b).

Besides, three different search engines were compared for the analysis of O-GlcNAc proteomics data files (Figure 1b). The resulting PSMs for all peptides were further processed by HexNAcQuest, excluding O-GalNAc peptides based on the oxonium ion patterns.^47^ The lists of PSMs for O-GlcNAc peptides (O-GlcNAc PSMs), O-GlcNAcylated proteins (containing unambiguous sites and/or ambiguous sites), and unambiguously identified sites are shown in Tables S1, S2, and S3, respectively.

### Enrichment of O-GlcNAc Peptides Before or After HPLC Fractionation

**Figure 2:**
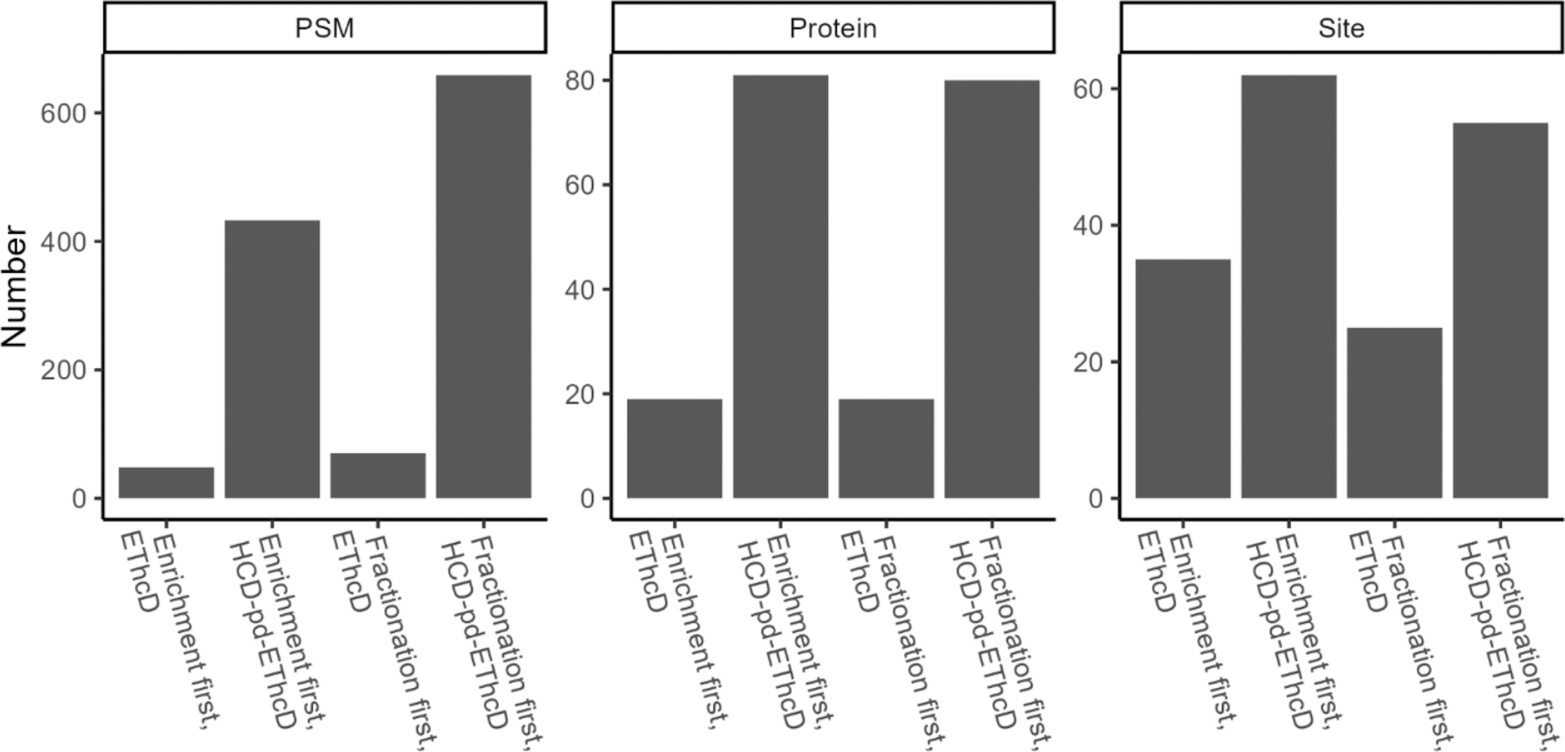
Comparison of O-GlcNAc PSMs, O-GlcNAc proteins, and unambiguous O-GlcNAc sites identified by using antibody-based enrichment (with the PTMScan O-GlcNAc [GlcNAc-S/T] motif kit), either EThcD mass spectrometry or HCD-pd-EThcD mass spectrometry, and Sequest HT-based data analysis for PANC-1 cell lysates.

By analyzing PANC-1 cell lysates with the O-GlcNAc antibody-based enrichment, we compared two analytical workflows: (1) enrich O-GlcNAc peptides from the peptide mixture and then fractionate into multiple fractions and (2) fractionate the peptide mixture and then enrich each fraction. To obtain site-specific information, we analyzed enriched peptides with either EThcD or HCD-pd-EThcD mass spectrometry. The resulting data files were processed with Sequest HT embedded in Proteome Discoverer v2.4. As shown in Figure 2, in comparison to the fractionation followed by enrichment approach, enrichment followed by fractionation yielded more O-GlcNAc sites (62 vs 55 in HCD-pd-EThcD, 35 vs 25 in EThcD), suggesting that the fractionation decreased the complexity of the eluates after enrichment and benefited the mass spectrometry detection and localization of modification sites. Interestingly, the number of identified O-GlcNAc proteins was almost the same between the two approaches.

**Figure 3:**
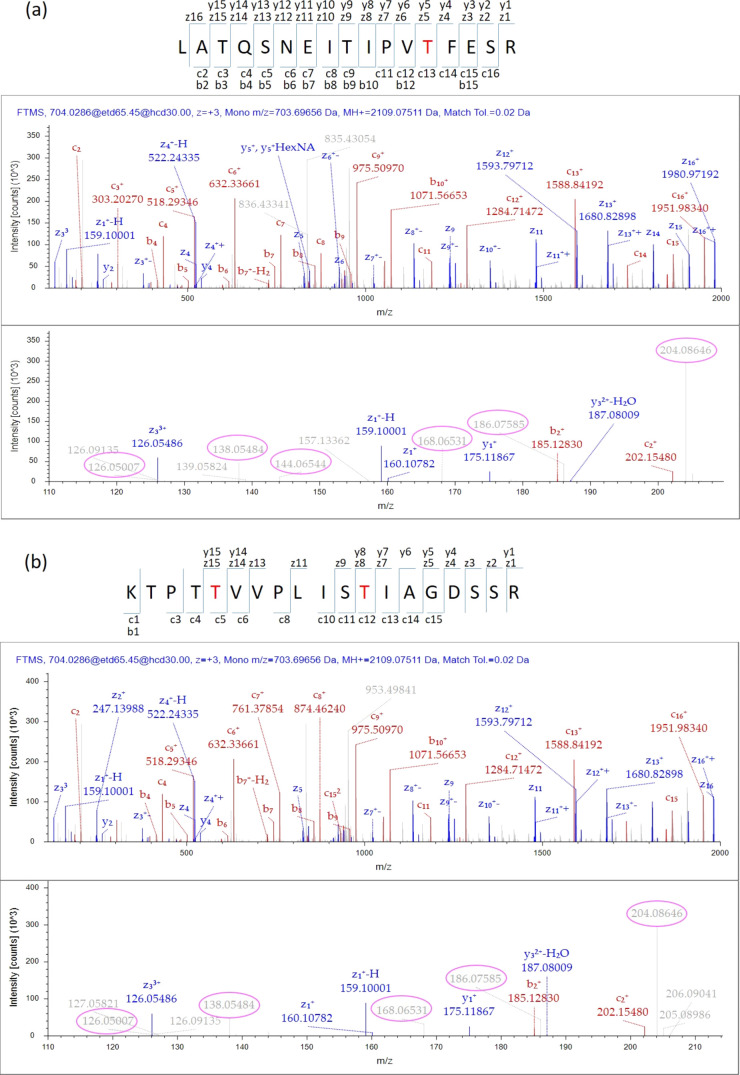
Mass spectra of peptides with (a) one O-GlcNAc site (i.e., T184 on HSPB1) and (b) two O-GlcNAc sites (i.e., T111 and T118 on VE2F) identified by using antibody-based enrichment, HCD-pd-EThcD mass spectrometry, and Sequest HT-based data analysis. The modified amino acids were shown in red, with matched fragments labeled. The corresponding HexNAc oxonium ions were circled out in the enlarged panels (bottom).

Regardless of the enrichment/fractionation approaches used, HCD-pd-EThcD mass spectrometry produced much more O-GlcNAc PSMs than EThcD (Figure 2; 433 vs 48; 659 vs 70 PSMs). Moreover, in comparison to EThcD, HCD-pd-EThcD yielded >3-fold more O-GlcNAc proteins and ∼2-fold more O-GlcNAc sites. These results show that HCD-pd-EThcD, in which oxonium ions of GlcNAc were used to trigger EThcD, is a preferred fragmentation method for improved O-GlcNAc identification. The representative MS/MS spectra of a peptide with one O-GlcNAc site (on heat shock protein β-1, HSPB1) and another peptide with two O-GlcNAc sites (on protein vascular endothelial zinc finger 1, VE2F) identified from HCD-pd-EThcD are illustrated in Figure 3a,3b, respectively.

Given that the ‘enrichment followed by fractionation’ approach yielded more O-GlcNAc sites, it was then used for the analytical workflows with other enrichment methods/materials. As HCD-pd-EThcD mass spectrometry produced remarkably better identification of O-GlcNAc (in comparison to EThcD), it was adopted in the following experiments.

### Evaluation of Affinity Enrichment Methods/Materials

**Figure 4:**
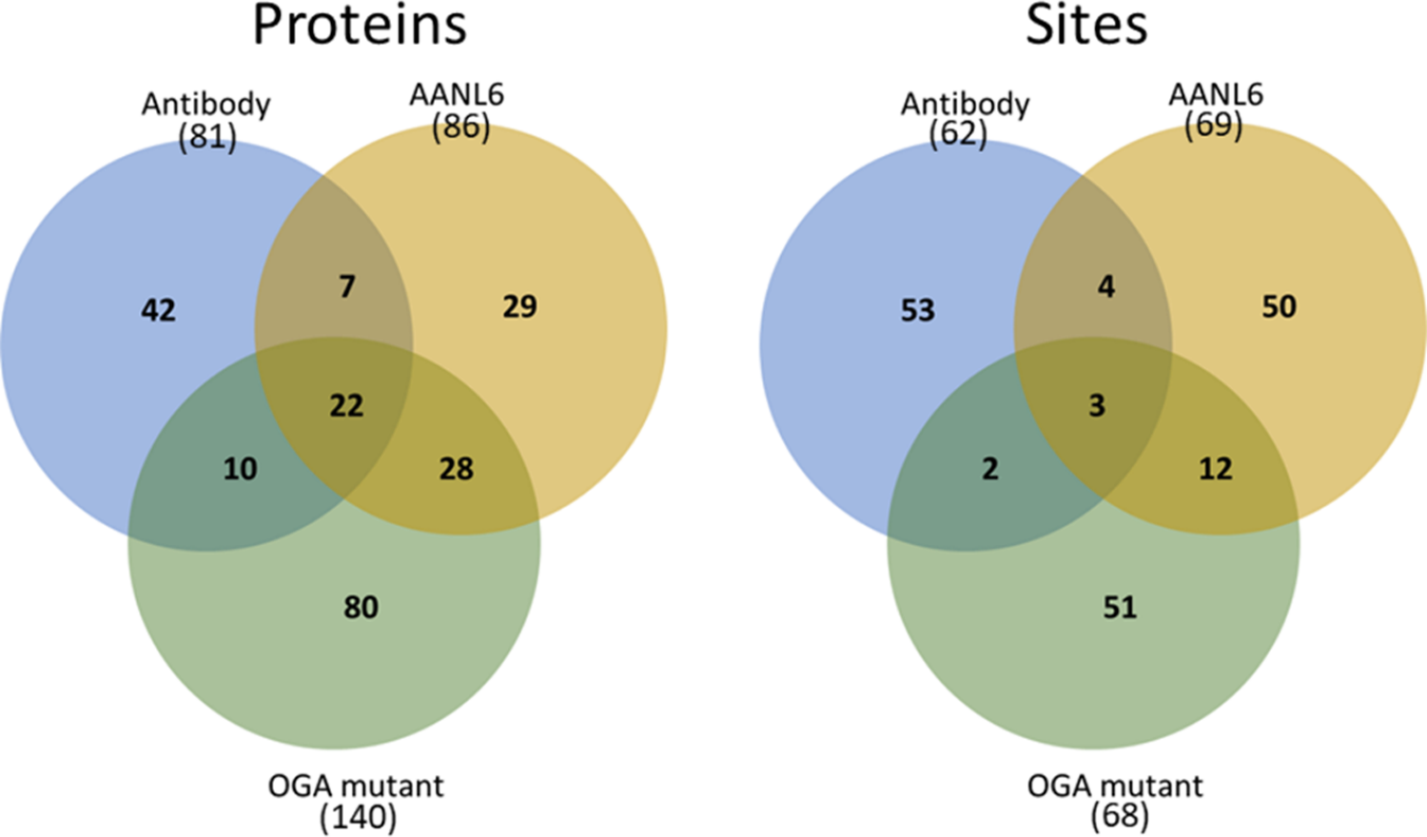
Comparison of O-GlcNAc proteins and unambiguous O-GlcNAc sites identified by using different enrichment methods/materials, HCD-pd-EThcD mass spectrometry, and Sequest HT-based data analysis.

Equal amounts of PANC-1 peptides were enriched by the immobilized O-GlcNAc antibody, AANL6, and the OGA mutant (CpOGA^D298N^), followed by fractionation into 12 fractions. The O-GlcNAc peptides were then analyzed by HCD-pd-EThcD mass spectrometry, with the resulting data files processed by Sequest HT embedded in Proteome Discoverer v2.4. As shown in Figure 4, the OGA mutant-based enrichment yielded 140 O-GlcNAc proteins (containing unambiguous sites and/or ambiguous sites), which is substantially more than those obtained with the antibody and AANL6 (81 and 86, respectively). In addition, most of these proteins were found only in the OGA mutant-based enrichment. Interestingly, although the three methods/materials identified similar numbers of unambigous O-GlcNAc sites (∼65), only three of them overlapped by the three methods. These results indicate that the three methods have distinct enrichment mechanisms, which may lead to strong complementarity for site-specific O-GlcNAc proteomics. Indeed, it appears that the PTMScan O-GlcNAc [GlcNAc-S/T] motif kit contains several immobilized antibodies aiming at specific motifs of O-GlcNAc peptides. In contrast, the AANL6 is a mutated lectin with high specificity for the terminal GlcNAc glycans, whereas the inactive OGA mutant retains the ability of the catalytically active enzyme to bind to the O-GlcNAcylated peptides. Thus, the distinct interaction mechanisms of these materials may contribute to identifying only a subpopulation of the O-GlcNAc proteome. Since a single affinity enrichment material with high selectivity toward the O-GlcNAc peptides is not currently available, a combination of different methods shall be beneficial for enhanced coverage of the O-GlcNAc proteome.

To further figure out the reason(s) for the low overlap of O-GlcNAc identification by the three enrichment methods, we assessed the reproducibility of the enrichment methods. This was exemplified by evaluating the identification of O-GlcNAc peptides after triplicate enrichment with AANL6 immobilized beads, one material that showed performance similar to that of the other two methods described. Instead of using complex samples (cell lysates) which need fractionation to reduce complexity before nanoHPLC-MS/MS analysis, we used synthetic peptides to estimate the repeatability of the enrichment methods. In brief, we spiked the mixture of 100 standard O-GlcNAcylated peptides (SpikeMix PTM-Kit 57 from JPT, 0.5 pmol per peptide) in 1 μg of yeast digest and performed the one-pot enrichment by using AANL6 immobilized beads. The enriched peptides were then subjected to HCD-pd-EThcD mass spectrometry, followed by Sequest HT analysis. Among the 24 O-GlcNAc peptides identified from three parallel enrichments, at least 12 (50%) were identified by duplicate enrichments and 6 (25%) by all three enrichments (Figure S1). This representative result demonstrates good repeatability of an individual enrichment method, further indicating that the low overlaps of the three enrichment methods mainly result from their distinct mechanisms.

We also evaluated the specificity of the enrichment methods. The O-GlcNAc peptide ratios identified from the three parallel enrichments with AANL6 immobilized beads for the spiked yeast digests were 9.5, 12.4, and 10.0%. Next, the specificity of the different enrichment materials for complex samples was evaluated by comparing percentages of HexNAc-modified peptides in total peptides identified from PANC-1 cell lysates (Figure S2). It appeared that antibody enrichment provides the best specificity (∼6.5%). However, the average specificity was relatively low (<5%), rendering the presence of a high percentage of nonmodified peptides after enrichment. This is consistent with our finding that extensive fractionation after enrichment can help to decrease the complexity and benefit the O-GlcNAc identification. The overall low enrichment specificity of these affinity-based methods might be due to their relatively low affinity toward O-GlcNAc peptides in complex samples.

### Evaluation of Data Analysis Packages

**Figure 5:**
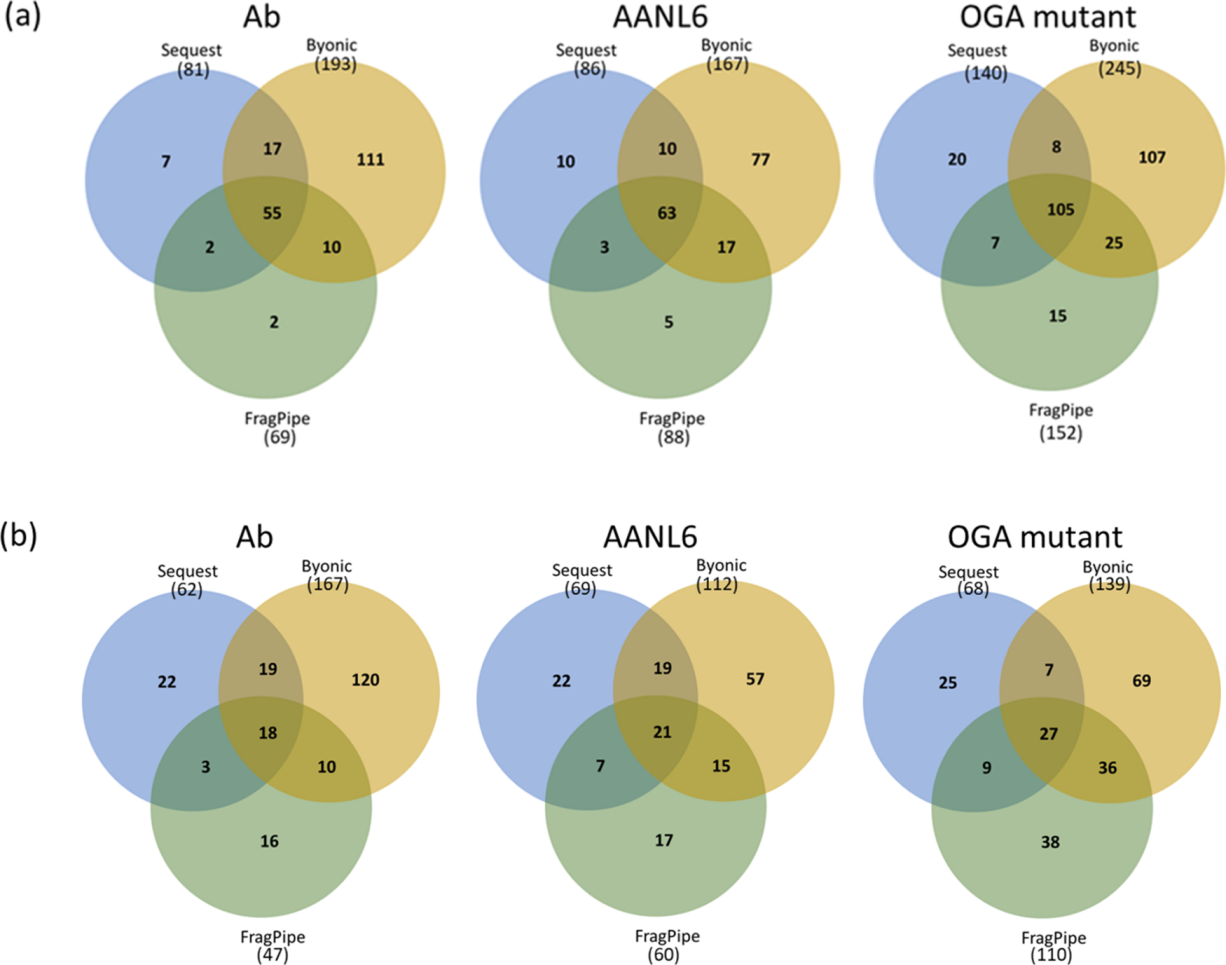
Overlap of O-GlcNAc proteins (a) and unambiguous O-GlcNAc sites (b) identified by different search engines. The PANC-1 peptide mixture was enriched by different methods/materials, followed by high-pH RPLC fractionation and then HCD-pd-EThcD mass spectrometry.

O-GlcNAc proteomics data analysis is not trivial. We analyzed the raw data files acquired after enrichment by three different search engines (i.e., Sequest HT, Byonic, and FragPipe) to identify the O-GlcNAcylation with similar parameters. In comparison, Byonic appeared to identify substantially more O-GlcNAc proteins from the antibody-based enriched sample, i.e., ∼2.4-fold (193 vs 81) and 2.8-fold (193 vs 69) higher than those identified by Sequest HT and FragPipe, respectively (Figure 5a). Up to 2-fold improvement in terms of the number of O-GlcNAc proteins was observed for the AANL6-based enrichment. Furthermore, Byonic appeared to identify substantially more O-GlcNAc sites after the antibody-based enrichment, i.e., ∼ 2.7-fold (167 vs 62) and 3.6-fold (167 vs 47) higher than those identified by Sequest HT and FragPipe, respectively (Figure 5b). Up to 2-fold improvement for the number of O-GlcNAc sites was also observed after AANL6-based enrichment. In comparison to Sequest HT and FragPipe, fewer than 2-fold increased numbers of O-GlcNAc proteins/sites were identified by Byonic for the OGA mutant-based enrichment. However, the OGA mutant-based enrichment coupled with Byonic analysis yielded the highest number of O-GlcNAc proteins (245) identified. While the highest number of O-GlcNAc sites (167) were obtained by the antibody-based enrichment coupled with Byonic analysis. Clearly, these results demonstrate the overall superior performance of the Byonic algorithm, regardless of the samples that were analyzed. Of note, we recently proposed a hydrophilicity chromatography method by developing nitro-oxide-grafted affinity nanospheres for suspension-mode enrichment (in comparison to the enrichment by using packed columns), in which the enriched peptides were also analyzed by a similar approach (i.e., fractionation followed by HCD-pd-EThcD mass spectrometry and data processing by Sequest HT embedded in Proteome Discoverer v2.4).^38^ Given that 230 O-GlcNAc sites were unambiguously identified from 5 mg of total protein lysates of PANC-1 cells as the starting material,^38^ the three affinity enrichment methods evaluated herein may have a bit lower performance.

Last but not least, if we combine the results from the three search engines, the number of O-GlcNAc proteins and sites identified only by one of the three enrichment methods was 72 and 89%, respectively (Figure S3), further indicating the high complementarity among different enrichment methods/materials. Collectively, these results suggest that a combination of the enrichment methods, combined with different data analysis tools, would contribute to a greater depth of O-GlcNAc proteomics.

### O-GlcNAcylation Identified in PANC-1 Cells

**Figure 6:**
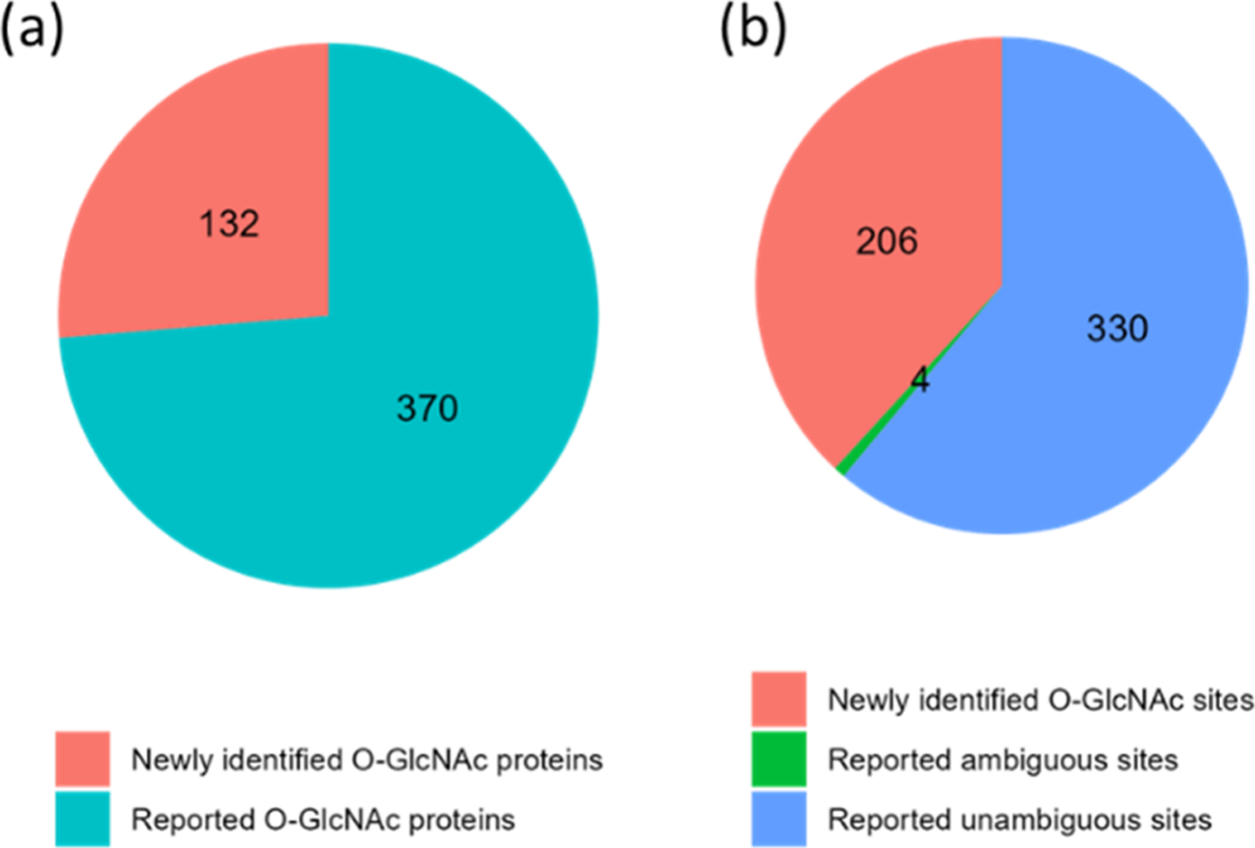
Comparison of (a) O-GlcNAc proteins and (b) unambiguously identified O-GlcNAc sites with those reported in the O-GlcNAcAtlas (version 3.0).

The systematic comparison of the analytical techniques in this study yielded a total of 502 O-GlcNAc proteins, with 540 unambiguous O-GlcNAc sites identified from PANC-1 cells. As shown in Figure 6, 132 (26%) proteins and 206 (38%) sites were not reported previously according to the O-GlcNAcAtlas (version 3.0), a database of experimentally identified O-GlcNAc sites and proteins (searched on April 15, 2024).^57^

The GO biological process analysis showed that the O-GlcNAc proteins are highly enriched in intermediate filament cytoskeleton organization and activation of the innate immune response, with a large portion of proteins involved in processes related to DNA and RNA (Figure S4a). The molecular functions of the O-GlcNAc proteins appear to be highly involved in various bindings, e.g., TPR domain binding, profilin binding, and nuclear localization sequence binding (Figure S4b).

## Conclusions

In this study, we performed a head-to-head evaluation of different methods, especially affinity enrichment methods/materials (i.e., antibody, lectin AANL6, and an OGA mutant) for O-GlcNAc proteomics, using PANC-1 cells. Instead of the dominant advantages of one method over the other, the methods show great complementarity for O-GlcNAc proteins/sites identified. Besides the enrichment methods, complementarity is also observed between data analysis tools (i.e., Sequest HT, Byonic, and FragPipe). Our work suggests that a combination of the different enrichment methods, by integration with different data analysis tools, would contribute to enhanced coverage of O-GlcNAc proteins and sites.

## Data Availability

Data Availability All mass spectrometry data files have been deposited to the MassIVE data repository (ftp://massive.ucsd.edu/v07/MSV000094604/).

## Supporting Information Available

Overlap of O-GlcNAc peptides identified by three parallel enrichments from the yeast digest spiked with standard O-GlcNAc peptides (Figure S1); ratios of distinct peptide sequences with HexNAc modification in all peptides identified from PANC-1 lysate samples (Figure S2); total O-GlcNAc proteins and unambiguous O-GlcNAc sites identified by a combination of Sequest HT, Byonic, and FragPipe (Figure S3); and GO analysis regarding biological process and molecular function of highly enriched O-GlcNAc proteins identified from PANC-1 cells (Figure S4) (PDF)
Lists of O-GlcNAc PSMs identified by different approaches (Table S1) (XLSX)
List of O-GlcNAcylated proteins identified by different approaches (Table S2) (XLSX)
List of unambiguous O-GlcNAc sites identified by different approaches (Table S3) (XLSX)

## References

[ref1] Hart G. W.; Housley M. P.; Slawson C. Cycling of O-Linked Beta-N-Acetylglucosamine on Nucleocytoplasmic Proteins. Nature 2007, 446 (7139), 1017–1022. 10.1038/nature05815. 17460662

[ref2] Hart G. W.; Slawson C.; Ramirez-Correa G.; Lagerlof O. Cross Talk Between O-GlcNAcylation and Phosphorylation: Roles in Signaling, Transcription, and Chronic Disease. Annu. Rev. Biochem. 2011, 80, 825–858. 10.1146/annurev-biochem-060608-102511. 21391816 PMC3294376

[ref3] Bond M. R.; Hanover J. A. O-GlcNAc Cycling: A Link Between Metabolism and Chronic Disease. Annu. Rev. Nutr. 2013, 33, 205–229. 10.1146/annurev-nutr-071812-161240. 23642195 PMC10483992

[ref4] Yang X.; Qian K. Protein O-GlcNAcylation: Emerging Mechanisms and Functions. Nat. Rev. Mol. Cell Biol. 2017, 18 (7), 452–465. 10.1038/nrm.2017.22. 28488703 PMC5667541

[ref5] Chatham J. C.; Zhang J.; Wende A. R. Role of O-Linked N-Acetylglucosamine Protein Modification in Cellular (Patho)Physiology. Physiol. Rev. 2021, 101 (2), 427–493. 10.1152/physrev.00043.2019. 32730113 PMC8428922

[ref6] Ma J.; Hou C.; Wu C. Demystifying the O-GlcNAc Code: A Systems View. Chem. Rev. 2022, 122 (20), 15822–15864. 10.1021/acs.chemrev.1c01006. 35302357

[ref7] Ma J.; Hart G. W. O-GlcNAc Profiling: From Proteins to Proteomes. Clin. Proteomics 2014, 11 (1), 8 10.1186/1559-0275-11-8. 24593906 PMC4015695

[ref8] Maynard J. C.; Chalkley R. J. Methods for Enrichment and Assignment of N-Acetylglucosamine Modification Sites. Mol. Cell. Proteomics 2021, 20, 100031 10.1074/mcp.R120.002206. 32938750 PMC8724609

[ref9] Xu S.; Sun F.; Tong M.; Wu R. MS-Based Proteomics for Comprehensive Investigation of Protein O-GlcNAcylation. Mol. Omics 2021, 17 (2), 186–196. 10.1039/D1MO00025J. 33687411

[ref10] Ma J.; Wu C.; Hart G. W. Analytical and Biochemical Perspectives of Protein O-GlcNAcylation. Chem. Rev. 2021, 121 (3), 1513–1581. 10.1021/acs.chemrev.0c00884. 33416322

[ref11] Wells L.; Vosseller K.; Cole R. N.; Cronshaw J. M.; Matunis M. J.; Hart G. W. Mapping Sites of *O*-GlcNAc Modification Using Affinity Tags for Serine and Threonine Post-Translational Modifications. Mol. Cell. Proteomics 2002, 1 (10), 791–804. 10.1074/mcp.M200048-MCP200. 12438562

[ref12] Ramirez-Correa G. A.; Ma J.; Slawson C.; Zeidan Q.; Lugo-Fagundo N. S.; Xu M.; Shen X.; Gao W. D.; Caceres V.; Chakir K.; DeVine L.; Cole R. N.; Marchionni L.; Paolocci N.; Hart G. W.; Murphy A. M. Removal of Abnormal Myofilament O-GlcNAcylation Restores Ca2+ Sensitivity in Diabetic Cardiac Muscle. Diabetes 2015, 64 (10), 3573–3587. 10.2337/db14-1107. 26109417 PMC4587639

[ref13] Vocadlo D. J.; Hang H. C.; Kim E.-J.; Hanover J. A.; Bertozzi C. R. A Chemical Approach for Identifying O-GlcNAc-Modified Proteins in Cells. Proc. Natl. Acad. Sci. U.S.A. 2003, 100 (16), 9116–9121. 10.1073/pnas.1632821100. 12874386 PMC171382

[ref14] Woo C. M.; Lund P. J.; Huang A. C.; Davis M. M.; Bertozzi C. R.; Pitteri S. J. Mapping and Quantification of Over 2000 O-Linked Glycopeptides in Activated Human T Cells with Isotope-Targeted Glycoproteomics (Isotag). Mol. Cell. Proteomics 2018, 17 (4), 764–775. 10.1074/mcp.RA117.000261. 29351928 PMC5880114

[ref15] Khidekel N.; Ficarro S. B.; Clark P. M.; Bryan M. C.; Swaney D. L.; Rexach J. E.; Sun Y. E.; Coon J. J.; Peters E. C.; Hsieh-Wilson L. C. Probing the Dynamics of O-GlcNAc Glycosylation in the Brain Using Quantitative Proteomics. Nat. Chem. Biol. 2007, 3 (6), 339–348. 10.1038/nchembio881. 17496889

[ref16] Wang Z.; Udeshi N. D.; O’Malley M.; Shabanowitz J.; Hunt D. F.; Hart G. W. Enrichment and Site Mapping of O-Linked N-Acetylglucosamine by a Combination of Chemical/Enzymatic Tagging, Photochemical Cleavage, and Electron Transfer Dissociation Mass Spectrometry. Mol. Cell. Proteomics 2010, 9 (1), 153–160. 10.1074/mcp.M900268-MCP200. 19692427 PMC2808261

[ref17] Alfaro J. F.; Gong C.-X.; Monroe M. E.; Aldrich J. T.; Clauss T. R. W.; Purvine S. O.; Wang Z.; Camp D. G.; Shabanowitz J.; Stanley P.; Hart G. W.; Hunt D. F.; Yang F.; Smith R. D. Tandem Mass Spectrometry Identifies Many Mouse Brain O-GlcNAcylated Proteins Including EGF Domain-Specific O-GlcNAc Transferase Targets. Proc. Natl. Acad. Sci. U.S.A. 2012, 109 (19), 7280–7285. 10.1073/pnas.1200425109. 22517741 PMC3358849

[ref18] Li J.; Li Z.; Duan X.; Qin K.; Dang L.; Sun S.; Cai L.; Hsieh-Wilson L. C.; Wu L.; Yi W. An Isotope-Coded Photocleavable Probe for Quantitative Profiling of Protein O-GlcNAcylation. ACS Chem. Biol. 2019, 14 (1), 4–10. 10.1021/acschembio.8b01052. 30620550

[ref19] Ma J.; Wang W.-H.; Li Z.; Shabanowitz J.; Hunt D. F.; Hart G. W. O-GlcNAc Site Mapping by Using a Combination of Chemoenzymatic Labeling, Copper-Free Click Chemistry, Reductive Cleavage, and Electron-Transfer Dissociation Mass Spectrometry. Anal. Chem. 2019, 91 (4), 2620–2625. 10.1021/acs.analchem.8b05688. 30657688 PMC6756848

[ref20] Xu S.; Sun F.; Wu R. A Chemoenzymatic Method Based on Easily Accessible Enzymes for Profiling Protein O-GlcNAcylation. Anal. Chem. 2020, 92 (14), 9807–9814. 10.1021/acs.analchem.0c01284. 32574038 PMC7437014

[ref21] Liu J.; Hao Y.; He Y.; Li X.; Sun D.; Zhang Y.; Yang P.-Y.; Chen X. Quantitative and Site-Specific Chemoproteomic Profiling of Protein O-GlcNAcylation in the Cell Cycle. ACS Chem. Biol. 2021, 16 (10), 1917–1923. 10.1021/acschembio.1c00301. 34161081

[ref22] Chen Y.; Tang F.; Qin H.; Yue X.; Nie Y.; Huang W.; Ye M. Endo-M Mediated Chemoenzymatic Approach Enables Reversible Glycopeptide Labeling for O-GlcNAcylation Analysis. Angew. Chem., Int. Ed. 2022, 61 (23), e202117849 10.1002/anie.202117849. 35289036

[ref23] Vosseller K.; Trinidad J. C.; Chalkley R. J.; Specht C. G.; Thalhammer A.; Lynn A. J.; Snedecor J. O.; Guan S.; Medzihradszky K. F.; Maltby D. A.; Schoepfer R.; Burlingame A. L. *O*-Linked *N*-Acetylglucosamine Proteomics of Postsynaptic Density Preparations Using Lectin Weak Affinity Chromatography and Mass Spectrometry. Mol. Cell. Proteomics 2006, 5 (5), 923–934. 10.1074/mcp.T500040-MCP200. 16452088

[ref24] Trinidad J. C.; Barkan D. T.; Gulledge B. F.; Thalhammer A.; Sali A.; Schoepfer R.; Burlingame A. L. Global Identification and Characterization of Both O-GlcNAcylation and Phosphorylation at the Murine Synapse. Mol. Cell. Proteomics 2012, 11 (8), 215–229. 10.1074/mcp.O112.018366. 22645316 PMC3412957

[ref25] Nagel A. K.; Schilling M.; Comte-Walters S.; Berkaw M. N.; Ball L. E. Identification of O-Linked N-Acetylglucosamine (O-GlcNAc)-Modified Osteoblast Proteins by Electron Transfer Dissociation Tandem Mass Spectrometry Reveals Proteins Critical for Bone Formation. Mol. Cell. Proteomics 2013, 12 (4), 945–955. 10.1074/mcp.M112.026633. 23443134 PMC3617341

[ref26] Xu S.-L.; Chalkley R. J.; Maynard J. C.; Wang W.; Ni W.; Jiang X.; Shin K.; Cheng L.; Savage D.; Hühmer A. F. R.; Burlingame A. L.; Wang Z.-Y. Proteomic Analysis Reveals O-GlcNAc Modification on Proteins with Key Regulatory Functions in Arabidopsis. Proc. Natl. Acad. Sci. U.S.A. 2017, 114 (8), E1536–E1543. 10.1073/pnas.1610452114. 28154133 PMC5338445

[ref27] Maynard J. C.; Fujihira H.; Dolgonos G. E.; Suzuki T.; Burlingame A. L. Cytosolic N-GlcNAc Proteins Are Formed by the Action of Endo-β-N-Acetylglucosaminidase. Biochem. Biophys. Res. Commun. 2020, 530 (4), 719–724. 10.1016/j.bbrc.2020.06.127. 32782141 PMC7508226

[ref28] Zhao P.; Viner R.; Teo C. F.; Boons G.-J.; Horn D.; Wells L. Combining High-Energy C-Trap Dissociation and Electron Transfer Dissociation for Protein O-GlcNAc Modification Site Assignment. J. Proteome Res. 2011, 10 (9), 4088–4104. 10.1021/pr2002726. 21740066 PMC3172619

[ref29] Lee A.; Miller D.; Henry R.; Paruchuri V. D. P.; O’Meally R. N.; Boronina T.; Cole R. N.; Zachara N. E. Combined Antibody/Lectin-Enrichment Identifies Extensive Changes in the O-GlcNAc Sub-Proteome Upon Oxidative Stress. J. Proteome Res. 2016, 15 (12), 4318–4336. 10.1021/acs.jproteome.6b00369. 27669760 PMC8132933

[ref30] Song H.; Ma J.; Bian Z.; Chen S.; Zhu J.; Wang J.; Huang N.; Yin M.; Sun F.; Xu M.; Pan Q. Global Profiling of O-GlcNAcylated and/or Phosphorylated Proteins in Hepatoblastoma. Signal Transduction Targeted Ther. 2019, 4 (1), 40 10.1038/s41392-019-0067-4. PMC6799812 31637018

[ref31] Burt R. A.; Dejanovic B.; Peckham H. J.; Lee K. A.; Li X.; Ounadjela J. R.; Rao A.; Malaker S. A.; Carr S. A.; Myers S. A. Novel Antibodies for the Simple and Efficient Enrichment of Native O-GlcNAc Modified Peptides. Mol. Cell. Proteomics 2021, 20, 100167 10.1016/j.mcpro.2021.100167. 34678516 PMC8605273

[ref32] Wang Z.; Pandey A.; Hart G. W. Dynamic Interplay between *O*-Linked *N*-Acetylglucosaminylation and Glycogen Synthase Kinase-3-Dependent Phosphorylation. Mol. Cell. Proteomics 2007, 6 (8), 1365–1379. 10.1074/mcp.M600453-MCP200. 17507370

[ref33] Jiang S.; Chen Y.; Wang M.; Yin Y.; Pan Y.; Gu B.; Yu G.; Li Y.; Wong B. H. C.; Liang Y.; Sun H. A Novel Lectin from *Agrocybe aegerita* Shows High Binding Selectivity for Terminal N-Acetylglucosamine. Biochem. J. 2012, 443 (2), 369–378. 10.1042/BJ20112061. 22268569 PMC3316157

[ref34] Su Y.; Ye X.; Xu B.; Li Y.; Yang Q.; Yu W.; Song J.; Guo C.; Wang X.; Hart G. W.; Sun H. CBS Homogenization Mutation Strategy Narrows the Glycan Binding Profile of a GlcNAc-Specific Lectin AANL. Glycobiology 2020, 30 (3), 159–173. 10.1093/glycob/cwz089. 31616917

[ref35] Mariappa D.; Selvan N.; Borodkin V.; Alonso J.; Ferenbach A. T.; Shepherd C.; Navratilova I. H.; vanAalten D. M. F. A Mutant O-GlcNAcase as a Probe to Reveal Global Dynamics of Protein O-GlcNAcylation during Drosophila Embryonic Development. Biochem. J. 2015, 470 (2), 255–262. 10.1042/BJ20150610. 26348912 PMC4941924

[ref36] Selvan N.; Williamson R.; Mariappa D.; Campbell D. G.; Gourlay R.; Ferenbach A. T.; Aristotelous T.; Hopkins-Navratilova I.; Trost M.; van Aalten D. M. F. A Mutant O-GlcNAcase Enriches Drosophila Developmental Regulators. Nat. Chem. Biol. 2017, 13 (8), 882–887. 10.1038/nchembio.2404. 28604694 PMC7611224

[ref37] Shen B.; Zhang W.; Shi Z.; Tian F.; Deng Y.; Sun C.; Wang G.; Qin W.; Qian X. A Novel Strategy for Global Mapping of O-GlcNAc Proteins and Peptides Using Selective Enzymatic Deglycosylation, HILIC Enrichment and Mass Spectrometry Identification. Talanta 2017, 169, 195–202. 10.1016/j.talanta.2017.03.049. 28411811

[ref38] Wu C.; Shi S.; Hou C.; Luo Y.; Byers S.; Ma J. Design and Preparation of Novel Nitro-Oxide-Grafted Nanospheres with Enhanced Hydrogen Bonding Interaction for O-GlcNAc Analysis. ACS Appl. Mater. Interfaces 2022, 14 (42), 47482–47490. 10.1021/acsami.2c15039. 36240223 PMC9938961

[ref39] Singh C.; Zampronio C. G.; Creese A. J.; Cooper H. J. Higher Energy Collision Dissociation (HCD) Product Ion-Triggered Electron Transfer Dissociation (ETD) Mass Spectrometry for the Analysis of N-Linked Glycoproteins. J. Proteome Res. 2012, 11 (9), 4517–4525. 10.1021/pr300257c. 22800195

[ref40] Halim A.; Westerlind U.; Pett C.; Schorlemer M.; Rüetschi U.; Brinkmalm G.; Sihlbom C.; Lengqvist J.; Larson G.; Nilsson J. Assignment of Saccharide Identities through Analysis of Oxonium Ion Fragmentation Profiles in LC-MS/MS of Glycopeptides. J. Proteome Res. 2014, 13 (12), 6024–6032. 10.1021/pr500898r. 25358049

[ref41] Tabb D. L. The SEQUEST Family Tree. J. Am. Soc. Mass Spectrom. 2015, 26 (11), 1814–1819. 10.1007/s13361-015-1201-3. 26122518 PMC4607603

[ref42] Haynes P. A.; Aebersold R. Simultaneous Detection and Identification of O-GlcNAc-Modified Glycoproteins Using Liquid Chromatography–Tandem Mass Spectrometry. Anal. Chem. 2000, 72 (21), 5402–5410. 10.1021/ac000512w. 11080893

[ref43] Cao Q.; Yu Q.; Liu Y.; Chen Z.; Li L. Signature-Ion-Triggered Mass Spectrometry Approach Enabled Discovery of N- and O-Linked Glycosylated Neuropeptides in the Crustacean Nervous System. J. Proteome Res. 2020, 19 (2), 634–643. 10.1021/acs.jproteome.9b00525. 31875397 PMC7441070

[ref44] Bern M.; Kil Y. J.; Becker C. Byonic: Advanced Peptide and Protein Identification Software. Curr. Protoc. Bioinf. 2012, 40, 13–20. 10.1002/0471250953.bi1320s40. PMC3545648 23255153

[ref45] Kong A. T.; Leprevost F. V.; Avtonomov D. M.; Mellacheruvu D.; Nesvizhskii A. I. MSFragger: Ultrafast and Comprehensive Peptide Identification in Mass Spectrometry–Based Proteomics. Nat. Methods 2017, 14 (5), 513–520. 10.1038/nmeth.4256. 28394336 PMC5409104

[ref46] Mao J.; Zhu H.; Liu L.; Fang Z.; Dong M.; Qin H.; Ye M. MS-Decipher: A User-Friendly Proteome Database Search Software with an Emphasis on Deciphering the Spectra of O-Linked Glycopeptides. Bioinformatics 2022, 38 (7), 1911–1919. 10.1093/bioinformatics/btac014. 35020790

[ref47] Li W.; Hou C.; Li Y.; Wu C.; Ma J. HexNAcQuest: A Tool to Distinguish O-GlcNAc and O-GalNAc. J. Am. Soc. Mass Spectrom. 2022, 33 (10), 2008–2012. 10.1021/jasms.2c00172. 36122299

[ref48] HaileMariam M.; Eguez R. V.; Singh H.; Bekele S.; Ameni G.; Pieper R.; Yu Y. S-Trap, an Ultrafast Sample-Preparation Approach for Shotgun Proteomics. J. Proteome Res. 2018, 17 (9), 2917–2924. 10.1021/acs.jproteome.8b00505. 30114372

[ref49] Wu C.; Zhou S.; Mitchell M. I.; Hou C.; Byers S.; Loudig O.; Ma J. Coupling Suspension Trapping–Based Sample Preparation and Data-Independent Acquisition Mass Spectrometry for Sensitive Exosomal Proteomic Analysis. Anal. Bioanal. Chem. 2022, 414 (8), 2585–2595. 10.1007/s00216-022-03920-z. 35181835 PMC9101639

[ref50] Song J.; Liu C.; Wang X.; Xu B.; Liu X.; Li Y.; Xia J.; Li Y.; Zhang C.; Li D.; Sun H. O-GlcNAcylation Quantification of Certain Protein by the Proximity Ligation Assay and *Clostridium perfringen* OGAD298N(CpOGAD298N). ACS Chem. Biol. 2021, 16 (6), 1040–1049. 10.1021/acschembio.1c00185. 34105348

[ref51] Ma J.; Hart G. W. Analysis of Protein O-GlcNAcylation by Mass Spectrometry. Curr. Protoc. Protein Sci. 2017, 87 (1), 24.10.1–24.10.16. 10.1002/cpps.24. PMC5300742 28150883

[ref52] Wickham H.; Averick M.; Bryan J.; Chang W.; McGowan L.; François R.; Grolemund G.; Hayes A.; Henry L.; Hester J.; Kuhn M.; Pedersen T.; Miller E.; Bache S.; Müller K.; Ooms J.; Robinson D.; Seidel D.; Spinu V.; Takahashi K.; Vaughan D.; Wilke C.; Woo K.; Yutani H. Welcome to the Tidyverse. J. Open Source Software 2019, 4 (43), 1686 10.21105/joss.01686.

[ref53] Dominik R.; Michal B.; Laura B. Tidysq: Tidy Processing and Analysis of Biological Sequences, 2022. https://CRAN.R-project.org/package=tidysq.

[ref54] Omar W. Ggseqlogo: A “ggplot2” Extension for Drawing Publication-Ready Sequence Logos, 2017. https://CRAN.R-project.org/package=ggseqlogo.

[ref55] Chen H.; Boutros P. C. VennDiagram: A Package for the Generation of Highly-Customizable Venn and Euler Diagrams in R. BMC Bioinf. 2011, 12 (1), 35 10.1186/1471-2105-12-35. PMC3041657 21269502

[ref56] Sherman B. T.; Hao M.; Qiu J.; Jiao X.; Baseler M. W.; Lane H. C.; Imamichi T.; Chang W. DAVID: A Web Server for Functional Enrichment Analysis and Functional Annotation of Gene Lists (2021 Update). Nucleic Acids Res. 2022, 50 (W1), W216–W221. 10.1093/nar/gkac194. 35325185 PMC9252805

[ref57] Ma J.; Li Y.; Hou C.; Wu C. O-GlcNAcAtlas: A Database of Experimentally Identified O-GlcNAc Sites and Proteins. Glycobiology 2021, 31 (7), 719–723. 10.1093/glycob/cwab003. 33442735 PMC8351508

