# Supplementary material for: Systematic Evaluation of Affinity Enrichment Methods for O-GlcNAc Proteomics
Source: J Proteome Res. 2024 Sep 20;23(10):4422–32. doi: 10.1021/acs.jproteome.4c00388 (PMC11459509; doi:10.1021/acs.jproteome.4c00388)
Supplement: Supplementary file 1 — pr4c00388_si_001.pdf [file pr4c00388_si_001.pdf]

Supporting Information

Systematic evaluation of affinity enrichment methods for O-GlcNAc proteomics

Chunyan Hou,[1] Ci Wu,[1] Zichun Wu,[2] Yifan Cheng,[1] Weiyu Li,[1,3] Hui Sun,[4] Junfeng Ma[1,*]

[1] Department of Oncology, Lombardi Comprehensive Cancer Center, Georgetown University Medical Center, Washington DC 20007, USA

[2] Information Science and Technology College, Dalian Maritime University, Dalian 116026, China.

[3] Department of Applied Mathematics and Statistics, Johns Hopkins University, Baltimore, MD, 21218, USA

[4] Department of Biochemistry, College of Life Sciences, Wuhan University, Wuhan 430072, China

[*] To whom correspondence should be addressed: Tel: +1-202-6873802; e-mail: junfeng.ma@georgetown.edu; ORCID: 0000-0002-5183-5425

Table of Contents

**Description**	**Page No.**
**Figure S1.** Overlap of O-GlcNAc peptides identified by three enrichments from the yeast digest spiked with standard O-GlcNAc peptides.	S-2
**Figure S2.** Ratios of distinct peptide sequences with HexNAc modification in all peptides identified from PANC-1 lysate samples.	S-3
**Figure S3**. Total O-GlcNAc proteins and unambiguous O-GlcNAc sites identified by a combination of Sequest HT, Byonic, and FragPipe.	S-4
**Figure S4**. GO analysis regarding (a) biological process and (b) molecular function of highly enriched O-GlcNAc proteins identified from PANC-1 cells.	S-5
**Table S1.** Lists of O-GlcNAc PSMs identfied by different approaches.	
**Table S2**. List of O-GlcNAcylated proteins identified by different approaches.	
**Table S3**. List of unambiguous O-GlcNAc sites identified by different approaches.	

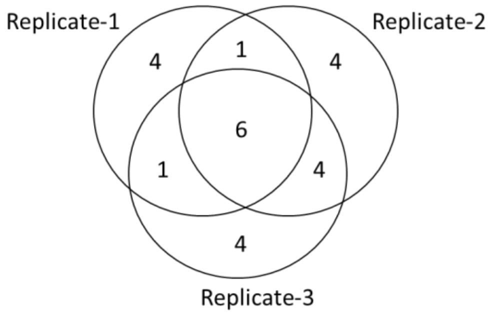

Figure S1. Overlap of O-GlcNAc peptides identified by three parallel enrichments from the yeast digest spiked with standard O-GlcNAc peptides. The peptide mixture was enriched by AANL6 immobilized beads and analyzed with HCD-pd-EThcD mass spectrometry followed by Sequest HT.

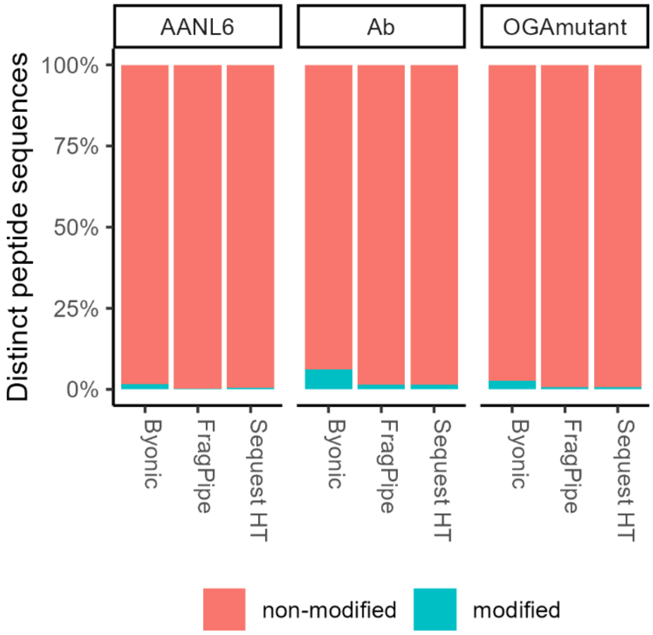

Figure S2. Ratios of distinct peptide sequences with HexNAc modification in all peptides identified from PANC-1 lysate samples. The peptide mixture was enriched by different materials followed by high pH RPLC fractionation and HCD-pd-EThcD mass spectrometry.

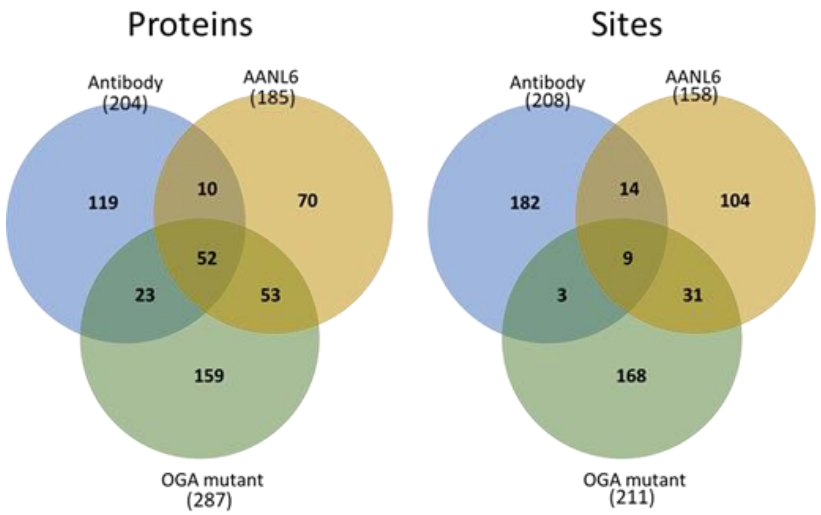

Figure S3. Total O-GlcNAc proteins and unambiguous O-GlcNAc sites identified by a combination of Sequest HT, Byonic, and FragPipe. The PANC-1 peptide mixture was enriched by different materials followed by high-pH RPLC fractionation, with HCD-pd-EThcD mass spectrometry used for site mapping.

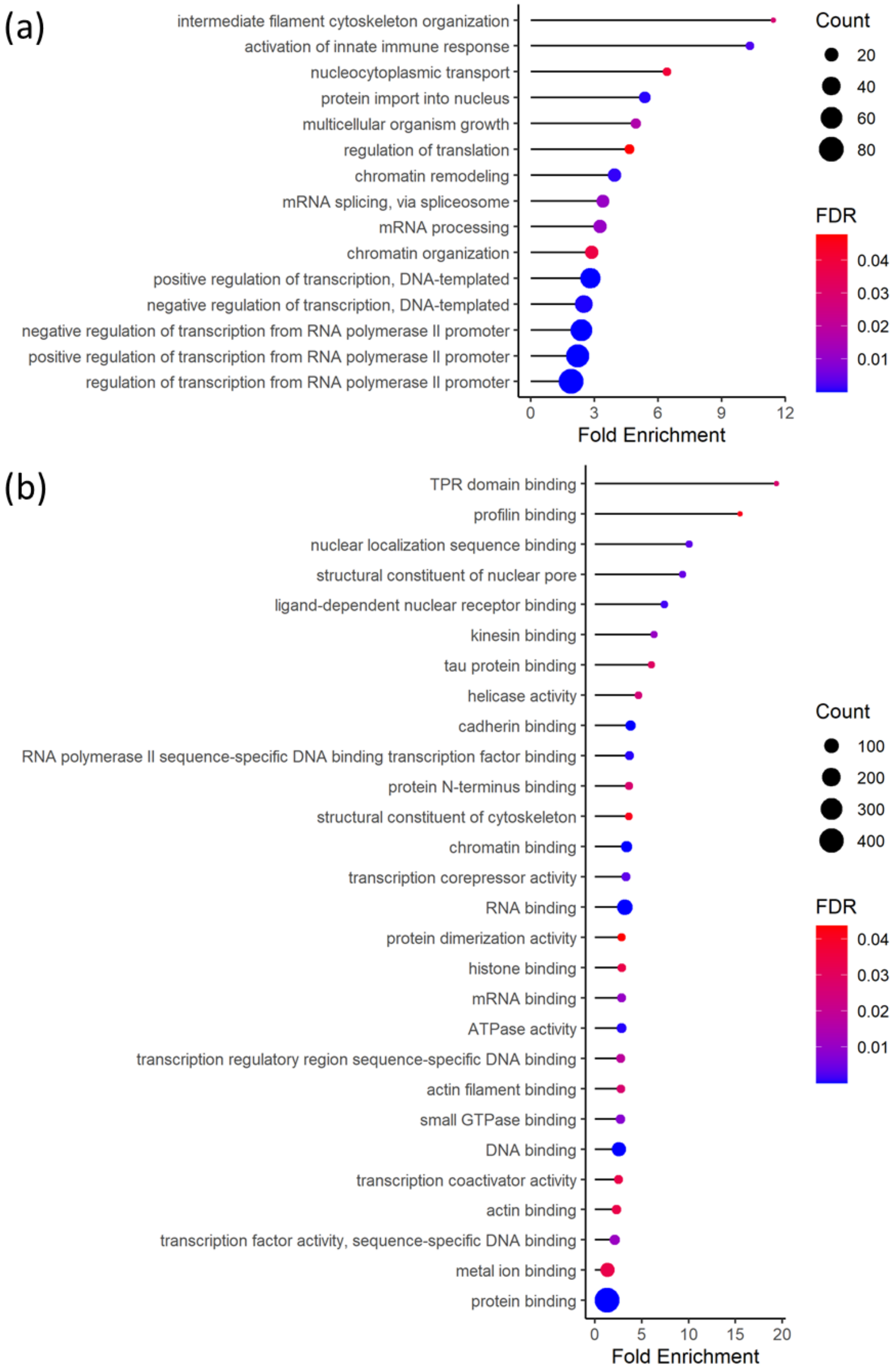

Figure S4. GO analysis regarding (a) biological process and (b) molecular function of highly enriched O-GlcNAc proteins identified from PANC-1 cells.
